# The Effect of Posterior Tibial Slope on the Risk of Revision Surgery After Anterior Cruciate Ligament Reconstruction

**DOI:** 10.1177/03635465211054100

**Published:** 2021-11-18

**Authors:** Lene Dæhlin, Eivind Inderhaug, Torbjørn Strand, Anagha P. Parkar, Eirik Solheim

**Affiliations:** † Faculty of Medicine, University of Bergen, Bergen, Norway; ‡ Department of Clinical Medicine, Faculty of Medicine, University of Bergen, Bergen, Norway; § Department of Orthopedics, Haukeland University Hospital, Bergen, Norway; ‖ Department of Orthopedics, Haraldsplass Deaconess Hospital, Bergen, Norway; ¶ Department of Radiology, Haraldsplass Deaconess Hospital, Bergen, Norway; # Aleris Nesttun Hospital, Bergen, Norway; Investigation performed at Haraldsplass Deaconess Hospital, Bergen, Norway

**Keywords:** ACL, ACL reconstruction, ACL revision, posterior tibial slope

## Abstract

**Background:**

A significant proportion of patients undergoing anterior cruciate ligament (ACL) reconstruction (ACLR) later experience graft failure. Some studies suggest an association between a steep posterior tibial slope (PTS) and graft failure.

**Purpose:**

To examine the PTS in a large cohort of patients about to undergo ACLR and to determine whether a steep PTS is associated with later revision surgery.

**Study Design:**

Case-control study; Level of evidence, 3.

**Methods:**

A retrospective review of a cohort undergoing isolated ACLR between 2002 and 2012 (with 8-19 years of follow-up) was conducted. Preoperative sagittal radiographs of knees in full extension were used for measurements of the PTS. There were 2 independent examiners who performed repeated measurements to assess the reliability of the method. Statistical analyses were performed to compare the PTS in the groups with and without later revision surgery.

**Results:**

A total of 728 patients, with a mean age of 28 years at the time of surgery, were included. Overall, 10% (n = 76) underwent revision surgery during the observation period. The group of injured knees had a significantly steeper PTS compared with the group of uninjured knees (9.5° vs 8.7°, respectively; *P* < .05). The mean PTS in the no revision group was 9.5° compared with 9.3° in the revision group (not significant). Dichotomized testing of revision rates related to PTS cutoff values of ≥10°, ≥12°, ≥14°, ≥16°, and ≥18° showed no association of PTS steepness (not significant) to graft failure. Patients with revision were younger than the ones without (mean age, 24 ± 8 vs 29 ± 10 years, respectively) and had a shorter time from injury to ACLR (mean, 14 ± 27 vs 24 ± 44 months, respectively) as well as a smaller graft size (8.2 vs 8.4 mm, respectively; *P* = .040).

**Conclusion:**

The current study did not find any association between a steep PTS measured on lateral knee radiographs and revision ACL surgery. However, a steeper PTS was seen in the group of injured knees compared with the group of uninjured (contralateral) knees. Independent of the PTS, younger patients, those with a shorter time from injury to surgery, and those with a smaller graft size were found to undergo revision surgery more often.

An anterior cruciate ligament (ACL) tear is a common and serious injury affecting professional as well as recreational athletes. For many, a torn ACL requires reconstructive surgery to regain their desired level of physical activity. Unfortunately, some of these patients later experience graft ruptures or other problems that necessitate revision surgery. Studies have shown that revision surgery, generally, has inferior outcomes compared with primary ACL reconstruction (ACLR).^14,15,36,42^ Thus, there is a need to identify factors that can explain why some patients experience graft failure and, further, to explore if these factors can be altered to reduce this risk.

Several clinical studies have concluded that there is an association between a steep posterior tibial slope (PTS) and an ACL tear^4,34,37,46^ as well as graft failure after reconstruction.^5,16,17,22,25,39^ Patients with a PTS of ≥12° are, in some studies, defined as an at-risk group.^25,39^ However, other studies have failed to demonstrate an association between a steep PTS and ACL injuries^28,35^ or graft ruptures.^6,35^ In a study by Cooper et al,^6^ which included only young patients (<22 years at the time of reconstruction), no association between an increased medial or lateral PTS and the need for revision surgery was found. They did, however, find that more patients in the revision group had a lateral PTS of ≥12°.

Biomechanical studies on cadaveric knees have investigated how changes in the PTS may affect anterior tibial translation (ATT) and thereby the forces exerted on the ACL.^12,13,21,43^ Decreasing the PTS has been shown to decrease ATT^21,43^ and reduce the strain on the native ACL.^2,31,43^ Shelburne et al^31^ found a linear relationship between changes in the PTS, ATT, and force exerted on the ACL while standing or walking. However, these findings are not consistent throughout other studies. Giffin et al^13^ showed that increasing the PTS caused a significant increase in ATT under axial compression, but with no significant change in force exerted on the ACL. Further, Fening et al^12^ found no change in tibial translation after anterior opening wedge osteotomy; rather, they saw decreased ACL strain when increasing the PTS.

It has been suggested that slope-reducing osteotomy might protect the reconstructed ACL by lowering the forces exerted on the graft under axial loading.^9,21^ Although there is some evidence that a steep PTS is associated with graft failure, there is no common agreement on which degree of steepness represents an unacceptable risk for increased strain on the native ACL or its substitute graft.^41^

The purpose of the study was to examine the PTS in a large cohort of patients with a torn ACL and to determine whether a steep PTS (a slope greater than the mean of the cohort as a whole) is associated with a higher rate of revision surgery after ACLR. The null hypothesis was that the PTS is not associated with future revision of ACL-reconstructed knees. In addition, we wanted to examine if any lower limit of the PTS showing an association with higher revision rates could be established.

## Methods

### Patient Selection

**Figure 1.**
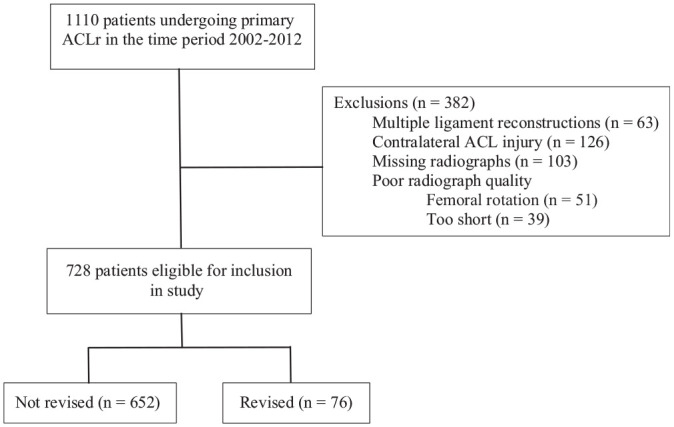
Flowchart of inclusion and exclusion of patients. ACL, anterior cruciate ligament; ACLR, ACL reconstruction.

All patients undergoing primary ACLR at Haraldsplass Deaconess Hospital between 2002 and 2012 were eligible for inclusion in the study. This period was chosen to allow the inclusion of a large enough cohort and to ensure sufficient follow-up time. Patients with concomitant injuries to other knee ligaments requiring surgery, ACL injuries to the contralateral knee, visible knee osteoarthritis on radiographs, and patients with missing or poor-quality preoperative radiographs were excluded (Figure 1). Descriptive patient data were extracted from a local knee database that collects information from every ACLR procedure conducted at our institution. Data on later revision surgery to the same knee were cross-checked with the National Knee Ligament Registry, making sure to include patients undergoing revision surgery at other institutions as well. The study was reviewed and approved by the regional ethical committee before data extraction (REK-ID 2019/1153).

### Surgical Technique

The vast majority of surgical procedures were performed, or supervised, by either of 2 senior surgeons (T.S.) at our institution. Different arthroscopically assisted surgical techniques were used through the course of the study period. Initially, a modified transtibial technique was applied (73%) before a transition to an “anatomic” technique (placing the femoral tunnel via an accessory anteromedial portal [27%]) was made in the latter part of the study period. All grafts were harvested from the ipsilateral knee, and a single-bundle graft configuration was used. A doubled hamstring tendon autograft was used in 96% of the patients, whereas 4% received a bone–patellar tendon–bone autograft. In patellar tendon reconstruction, a metal interference screw was used for both femoral and tibial fixation. In hamstring tendon reconstruction, using the transtibial approach, tibial post fixation was combined with either transfixation or cortical fixation. In the anatomic approach, a cortical flipping button was combined with a bioabsorbable interference screw.

### Radiographic Measurements

Measurements were made using the Impax picture archiving and communication system (Agfa HealthCare). Preoperative sagittal radiographs of the patients’ injured knee and (where available) contralateral uninjured knee in full extension were acquired for the study. These radiographs are routinely acquired preoperatively for all patients undergoing ACLR at our institution to obtain preoperative measurements of the intercondylar roof angle and to look for hyperextension in the injured knee compared with the uninjured knee. All measurements were made by an independent observer, a medical doctor (L.D.) trained by a musculoskeletal radiologist (A.P.P.), who was not involved in patient treatment and was blinded to which knee was injured and whether the patients had later undergone revision surgery. To calculate interrater reliability, 100 radiographs were randomly selected so that measurements could be repeated by the musculoskeletal radiologist. To calculate intrarater reliability, the main observer assessed these same 100 images a second time, blinded to the first assessments.

**Figure 2.**
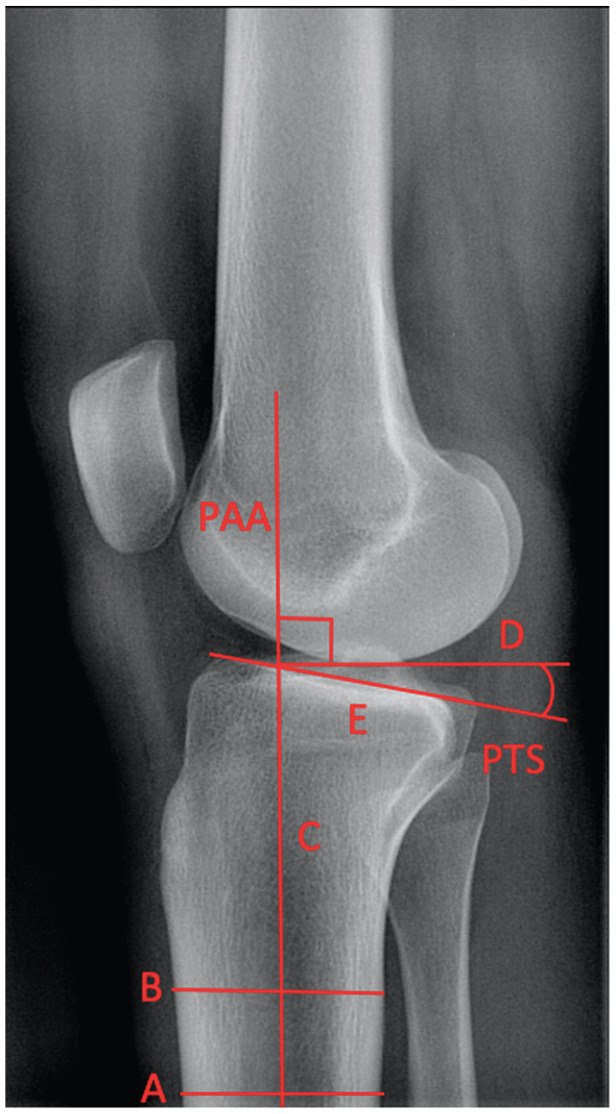
Measurement of the posterior tibial slope (PTS) on a lateral knee radiograph using the proximal anatomic axis (PAA) of the tibia as a reference line. The center point of lines *A* and *B* across the tibial shaft was used to determine the PAA of the tibia (line *C*). A perpendicular line to the PAA of the tibia was drawn (line *D*) as well as a line representing the PTS (line *E*) to allow measurements of the PTS in degrees as the angular difference between lines *D* and *E*.

The PTS was measured using the proximal anatomic axis (PAA) of the tibia^44^ as a reference for a line drawn between the anterior and posterior edges of the tibial plateau,^23^ representing the PTS (Figure 2). This axis has been found to best represent the sagittal mechanical axis of the tibia.^44^ To ensure correct measurements of the PAA of the tibia, only radiographs displaying a minimum length of 10 cm of the tibia were included. Further, radiographs showing <80% overlap of the femoral condyles were excluded to ensure that the degree of rotation of the tibia would not influence the measurements.^18,38^ A maximum of 5-mm vertical separation of the posterior tibial condyles was also used as an exclusion criterion to avoid knee rotation influencing the PTS measurements.

### Statistical Analysis

All statistical analyses were carried out using SPSS Statistics Version 25.0 for Mac (IBM). A level of significance of *P* < .05 was chosen a priori. The Shapiro-Wilk test was used to examine for normality of the data. One-way analysis of variance (ANOVA) was performed to compare differences in the PTS for the revision and no revision groups as well as for the groups of injured and uninjured knees. Repeated-measures ANOVA was used to investigate the within-participant difference in the PTS between the injured and uninjured knees of the same patient. Chi-square statistics were used to compare frequencies in the revision and no revision groups with respect to sex, right or left knee, type of graft, and graft size. Chi-square statistics were also used to compare frequencies in patients grouped by PTS values with respect to revision versus no revision and sex. Because of the nonnormality of data, the Mann-Whitney *U* test was used to compare differences between the revision and no revision groups in age at the time of surgery and time from injury to primary surgery. The intraclass correlation coefficient was calculated, using the absolute agreement 2-way random model for the determination of intrarater and interrater reliability for the PTS measurements. Binary logistic regression analysis was performed to investigate associations between revision and other patient variables. A sample size power analysis for a comparison of the PTS in Tables 1 and 2 (alpha = .05) showed a power of 98.5% and 100%, respectively, for detecting a minimal clinically important difference in the PTS of 1.5°± 0.5°.^29^

**Table 1:** Descriptive Data^*a*^

	No Revision (n = 652)	Revision (n = 76)	*P* Value
Sex, male:female, %	53:47	40:60	**.025**
Injured knee, right:left, %	53:47	47:53	.373
Type of graft, hamstring tendon:patellar tendon, %	96:4	96:4	.936
Surgical technique, transtibial:anteromedial, %	74:26	71:29	.159
Graft size, mm	8.4 ± 0.8	8.2 ± 0.8	**.040**
≥8:<8, %	95:5	85:15	**.001**
Age at primary reconstruction, y	29 ± 10	24 ± 8	**<.001**
Time from injury to primary reconstruction, mo	24 ± 44	14 ± 27	**.003**
PTS in injured knee, deg	9.5 ± 3.0	9.3 ± 3.0	.550

a Values are presented as mean ± SD unless otherwise indicated. Bold indicates *P* < .05. PTS, posterior tibial slope.

**Table 2:** Binary Logistic Regression Analysis for Association With Revision Surgery^*a*^

	Odds Ratio (95% CI)	*P* Value
Sex (female vs male)	1.177 (0.676-2.048)	.565
Age at time of primary reconstruction	0.956 (0.928-0.986)	**.004**
Time from injury to primary reconstruction	0.994 (0.983-1.005)	.271
Type of graft (patellar vs hamstring)	1.489 (0.375-5.914)	.572
Surgical technique (anteromedial vs transtibial)	1.222 (0.700-2.134)	.480
Graft size	0.725 (0.468-1.121)	.148
PTS	0.989 (0.912-1.074)	.798

a Nagelkerke *R*^2^ = 0.65. Bold indicates *P* < .05. PTS, posterior tibial slope.

## Results

### Demographic Data

A total of 728 patients were included in the study. Of these, 52% were men, and the mean age of the cohort at the time of surgery was 28 ± 10 years. Overall, 10% (n = 76) of the patients later underwent revision surgery. The mean time from injury to primary surgery was 23 ± 43 months, and the mean time from primary surgery to revision surgery was 41 ± 35 months. The median follow-up time after ACLR was 13 years (range, 8-19 years).

### Intrarater and Interrater Reliability

The intrarater and interrater reliability (intraclass correlation coefficient) for the radiographic measurements were calculated, showing good intrarater reliability (0.88; *P* < .001) and moderate interrater reliability (0.68; *P* < .001).^24^

### Demographics and Association With Revision Surgery

Patients who underwent revision in the study period were younger at the time of primary reconstruction compared with the ones without revision (mean, 24 ± 8 vs 29 ± 10 years, respectively; *P* < .001) and had a shorter time from the injury to primary reconstruction (mean, 14 ± 27 vs 24 ± 44 months, respectively; *P =* .003). A greater proportion of the patients undergoing revision surgery were women (60%; *P* = .025). There were no differences between the revision and no revision groups regarding the surgical technique applied or type of graft used (not significant). The graft size ranged between 7 and 12 mm (mean, 8.4 ± 0.7 mm) and was significantly smaller for the revision group compared with the no revision group (8.2 vs 8.4 mm, respectively; *P* = .040). Additionally, 6% (n = 43) of the grafts used were <8 mm in diameter. A higher proportion of the patients in the revision group had a graft size of <8 mm versus the patients in the no revision group (15% vs 5%, respectively; *P* = .001) (Table 1).

### Binary Logistic Regression Analysis Findings

The results of the binary logistic regression analysis between the revision and no revision groups and other independent variables are shown in Table 2. The model displayed statistically significant associations (chi-square [7] = 22.8; *P* = .002). The accuracy in classification was 89.7%. The results indicate that a lower age at the time of ACLR (odds ratio, 0.956) was associated with an increased rate of revision. None of the other variables showed significant differences between the groups.

### PTS in Study Population

We obtained PTS measurements from 728 injured knees as well as 499 uninjured knees. With these measurements, we conducted 2 separate analyses. First, we performed 1-way ANOVA using all of our PTS measurements to look for differences in means between the groups of uninjured and injured knees. The PTS of the group of injured knees ranged from 0.8° to 19.0°, with a mean of 9.5°± 3.0°, whereas the PTS measured in the group of uninjured knees ranged from 1.0° to 17.4°, with a mean of 8.7°± 3.0° (Table 3). This represents a statistically significant difference (*P* < .05). For 499 of the patients from whom we obtained PTS measurements of both the injured and the uninjured knees (patients with radiographs of both knees and without contralateral ACL injuries), we additionally performed repeated-measures ANOVA to measure the within-participant differences in the PTS. This showed that the patients’ injured knee had a significantly higher PTS value compared with that of the uninjured knee (9.4°± 3.0° vs 8.7°± 3.0°, respectively; *P* < .05) (Table 3). The mean within-participant difference in the PTS was 0.6°± 3.0° (*P* < .01), displaying not only a significant difference in the PTS between the groups of injured and uninjured knees but also a difference in the PTS between the injured and uninjured knees within each patient.

**Table 3:** PTS in Study Population^*a*^

	Uninjured (n = 499)	Injured (n = 728)	*P* Value
PTS	8.7 ± 3.0 (1.0-17.4)	9.5 ± 3.0 (0.8-19.0)	**<.001**
	Uninjured (n = 499)	Injured (n = 499)	*P* Value
Within-participant PTS	8.7 ± 3.0 (1.0-17.4)	9.4 ± 3.0 (0.8-16.8)	**<.001**

a Values are presented as mean ± SD (range) in degrees. Bold indicates *P* < .05. PTS, posterior tibial slope.

### Effect of PTS on Later Revision

The mean PTS in the no revision group was 9.5°± 3.0°, compared with 9.3°± 3.0° in the revision group (*P* = .550) (Table 1). Grouping patients dichotomously based on the PTS in their injured knee showed no association with undergoing later revision surgery for groups with PTS values of ≥10°, ≥12°, ≥14°, ≥16°, or ≥18° compared with the rest of the cohort (not significant for all comparisons) (Table 4). In the study population as a whole, 21% had a PTS of ≥12°. Significantly more men had a PTS of ≥14°, but a higher proportion of the women later underwent revision surgery compared with this proportion in the group of men (13% vs 8%, respectively; *P* = .025). Only 2 patients had extreme PTS values of ≥18°.

**Table 4:** Patients Grouped by PTS in Injured Knee^*a*^

	≥10° (n = 307)	≥12° (n = 149)	≥14° (n = 52)	≥16° (n = 12)	≥18° (n = 2)
No revision:revision	90:10	91:9	94:6	83:17	100:0
*P* value	.664	.466	.261	.468	.630
Male:female sex	52:48	58:42	69:31	58:42	50:50
*P* value	.740	.068	**.009**	.644	.961
Proportion of total cohort	42.2	20.5	7.1	1.7	0.3

a Values are presented as percentages. Bold indicates *P* < .05. PTS, posterior tibial slope.

## Discussion

The current study, investigating the PTS in 728 patients with an ACL tear, found no significant association between a steep PTS and later revision surgery. However, a steeper PTS was seen in the injured knees compared with the pooled group of uninjured knees in the between-participant analysis. Further, when comparing the within-participant difference in the PTS, the patients’ injured knee had a steeper PTS than the uninjured contralateral knee, suggesting that there also may be intraindividual anatomic differences in the PTS. The latter finding is rarely described but aligns with studies describing how ACL-injured knees have a steeper PTS compared with the knees of noninjured controls.^4,23,34,37,46^ It is important to notice that the differences in the measured PTS are small and may not be clinically relevant, even though there is a statistically significant difference. This implies that even though there is a significant difference in the PTS at a group level, the PTS assessed on radiographs may not be a suitable measure to predict the later need for revision at an individual level. To the best of our knowledge, the current study is the largest of its kind to investigate the PTS and its association with revision and adds to the discussion of whether slope-reducing osteotomy has a place in ACL revision surgery.

When comparing studies investigating the PTS, differences in the radiographic modality and method of measurement are important to consider. Short lateral knee radiographs were chosen for this study because of the availability, although studies have shown that PTS measurements on long lateral knee radiographs may be somewhat more accurate.^1,11^ However, Yoo et al^44^ demonstrated how the PAA of the tibia measured on short radiographs has good correlation with the sagittal mechanical axis of the tibia measured on long radiographs. Further, a study by Dean et al^8^ found no significant difference between the PTS measured on full-length tibial radiographs using the anatomic tibial axis compared with measurements using the PAA of the tibia on knee radiographs. As the current study used the same standardized protocol throughout the measurements, we believe, even if a potential overall skewness of data might exist, that the displayed differences (or lack thereof) between the groups are reliable. Using the longitudinal axis of the tibia (corresponding to the PAA of the tibia used in our study) has shown excellent agreement between reviewers,^23,46^ and this has been found to be the most reliable method for measuring the PTS on lateral knee radiographs.^46^ Therefore, we believe that the current exclusion of images with malrotation, and good agreement between repeat measurements, strengthens the current findings.

When using radiographs to measure the PTS, one cannot distinctly differentiate between the medial and lateral PTS, but the measurements are close to the real medial PTS.^19,20^ Based on our data, we therefore cannot draw any conclusions as to whether there is any difference in the relationship between revision surgery stratified by the lateral and medial PTS. This may be of interest if it is hypothesized that differences in the lateral and medial PTS create rotational forces within the joint that increase ACL strain.

The different methods of measuring the PTS make it challenging to compare across studies of the PTS. A systematic review by Wordeman et al^41^ showed that there is a vast disagreement regarding which values of the PTS should be considered “normal” and “at risk.” They found that differences between the values described as a normal PTS in different studies sometimes exceeded what is reported as the difference between controls and patients with ACL injuries. This is important to have in mind when discussing at-risk values of the PTS across radiologic modalities when treating ACL injuries, and these reported normal values can substantially differ. Comparing our measurements of the PTS with other studies that have also used lateral knee radiographs displays this fact. The mean PTS of uninjured and injured knees ranges in these studies from 5.6° to 9.9° and from 5.7° to 11.5°, respectively, with most standard deviations around 3.0°.^4,11,28,35,37,46^ Whether this discrepancy is caused by actual anatomic differences between populations or if it is because of variations in measurement methods is not possible to determine without an actual comparison of populations. Nevertheless, our findings are in agreement with these studies.

The current finding of no difference in the PTS between the revision and no revision groups is contrary to the majority of studies^5,16,22,25,39,45^ but is supported by some.^6,35^ Several authors have proposed a PTS of ≥12°, measured on knee radiographs, as a risk factor for ruptures of the ACL graft or revision.^25,39^ One study found that patients with a PTS of ≥12° had a 59% incidence of further ACL injuries compared with a 23% incidence in patients with a PTS of <12°, or a 5 times higher incidence of further injuries to the ACL when controlling for age and sex.^39^ On this rationale, Dejour et al^9^ suggested considering concomitant slope-reducing osteotomy when performing revision surgery in patients with a PTS of >12°. Our study did not find any association between revision and an increase in the PTS, even when dichotomizing patients with a PTS of ≥10°, ≥12°, ≥14°, ≥16°, or ≥18°. On the basis of the current results, one should therefore be careful using a PTS of ≥12° (measured on lateral knee radiographs) as a decision support when considering concomitant osteotomy in ACL surgery.

Even though several studies have performed slope-reducing osteotomy on cadaveric knees and found that this reduced ATT^21,43^ and ACL strain,^2,21^ these are studies that have been conducted in vitro without muscular loading, limiting their generalizability to a clinical setting. Shelburne et al^31^ used a computer model to calculate ATT, tibial shear force, and ACL forces in a healthy knee during activity. Their investigation of knee function related to changes in the PTS concluded that such effects can only be fully assessed under physiological conditions in which muscular forces are applied. Although decreasing the slope may be beneficial in the ACL-deficient knee, it is important to consider that an increased slope may protect the posterior cruciate ligament.^3^ It is therefore paramount, if one chooses to perform slope-reducing osteotomy, to find a middle ground that does not harm either the ACL or the posterior cruciate ligament.

Our results show that patients who underwent revision surgery were significantly younger at the time of ACLR. Other studies have similar findings,^10,27,30,33,39,40^ implying that young age is associated with an increased rate of graft failure. Maletis et al^27^ evaluated 21,304 patients who had undergone ACLR and found the youngest age group (<21 years) to have a 7.76 times higher risk of revision compared with the oldest age group (>40 years). A possible explanation for this finding can be that younger patients have greater demands on their postoperative knee function because of a more active lifestyle. A systematic review and meta-analysis by Wiggins et al^40^ found the greatest incidence of ACL reinjuries in young patients (<20-25 years) who returned to high-risk sports (23%). Most reinjuries occur within a few years after reconstruction.^30^ Snaebjornsson et al^33^ found the youngest age group at reconstruction (13-19 years) to have the highest risk of early revision (within 2 years after ACLR). Further studies should also explore whether these patients return to demanding activities too early after surgery.

Graft size has also been found to affect revision rates.^32^ Magnussen et al^26^ found that using a hamstring tendon graft of <8 mm in patients younger than 20 years was associated with higher revision rates. Our findings are similar, showing that a higher proportion of the patients in the revision group had received grafts of <8 mm and that the mean graft size was smaller in the revision group. Our regression analysis, however, found only age to be significant when comparing the revision and no revision groups. Another finding in the current study was a shorter time from injury to reconstructive surgery in those who later underwent revision. It is likely that the choice to undergo early surgery was related to a desire to return to high-risk sports and is therefore an indication of the level of activity. Unfortunately, measures of activity level were not available for the current analyses.

The present study has several limitations. First, we did not include a control group of uninjured participants. Second, we cannot account for all patients who experienced real graft failure, only those who underwent later revision surgery. Using revision to account for failure will underestimate the total number of patients with clinical failure.^7^ Third, considering that the inclusion period was relatively long, surgical techniques have developed and changed throughout this time, and this heterogeneity in surgical technique may come with subtle effects on outcomes. Finally, as always, human errors may occur, and there are small margins when measuring the PTS. This emphasizes the importance of not using the PTS as the sole factor when deciding whether to perform slope-reducing osteotomy.

## Conclusion

The results from the current study did not find any association between a steep PTS measured on lateral knee radiographs and revision ACL surgery. However, a steeper PTS was seen in the group of injured knees compared with the group of uninjured (contralateral) knees. As described in other studies, independent of the PTS, younger patients, those with a shorter time from injury to surgery, and those with a smaller graft size were found to undergo revision surgery more often.
